# Human Walking Direction Detection Using Wireless Signals, Machine and Deep Learning Algorithms

**DOI:** 10.3390/s23249726

**Published:** 2023-12-09

**Authors:** Hanan Awad Hassan Ali, Shinnazar Seytnazarov

**Affiliations:** 1 Faculty of Computer Science and Engineering, Innopolis University, 420500 Innopolis, Russia; h.ali@innopolis.university; 2 Faculty of Computers & Informatics, Suez Canal University, Ismailia 41522, Egypt

**Keywords:** human activity recognition, Wi-Fi signals, channel state information (CSI), walking direction detection

## Abstract

The use of wireless signals for device-free activity recognition and precise indoor positioning has gained significant popularity recently. By taking advantage of the characteristics of the received signals, it is possible to establish a mapping between these signals and human activities. Existing approaches for detecting human walking direction have encountered challenges in adapting to changes in the surrounding environment or different people. In this paper, we propose a new approach that uses the channel state information of received wireless signals, a Hampel filter to remove the outliers, a Discrete wavelet transform to remove the noise and extract the important features, and finally, machine and deep learning algorithms to identify the walking direction for different people and in different environments. Through experimentation, we demonstrate that our approach achieved accuracy rates of 92.9%, 95.1%, and 89% in detecting human walking directions for untrained data collected from the classroom, the meeting room, and both rooms, respectively. Our results highlight the effectiveness of our approach even for people of different genders, heights, and environments, which utilizes machine and deep learning algorithms for low-cost deployment and device-free detection of human activities in indoor environments.

## 1. Introduction

Human activity recognition (HAR) is an area of growing interest for researchers. Traditional approaches for HAR rely on cameras [1], radars [2,3,4,5], or wearable sensors [6,7]. However, camera-based approaches have limitations. First, the camera can work only in a line-of-sight (LOS) environment with sufficient lighting [8,9,10,11,12]. Second, it does not preserve privacy concerns [8,9,10,12,13,14,15,16,17,18,19,20,21,22,23,24,25]. Finally, it cannot track activities or gestures through walls. Wearable sensor-based approaches are uncomfortable because the users have to continuously carry wearable devices for human activity recognition [21,26]. To avoid the above problems, wireless signals are used to achieve HAR without user devices [15,21]. The human body reflects and scatters wireless signals, producing a unique pattern that can be used for identification. This is achieved by analyzing the received signal strength indicator (RSSI) or channel state information (CSI) of the wireless signals received by different antennas. We can use wireless signals to work in LOS or non-LOS (NLOS) environments even in dark conditions [12] and thus preserve users’ privacy [8,9,10,13,15,16,21,22,24,26,27,28].

One of the challenges in HAR systems is human walking direction detection (HWDD). HWDD can be used in various applications, including:

- In security systems, to track the movement of individuals in restricted areas and detect any unauthorized access or suspicious behavior.
- In healthcare, to monitor the movement of patients in hospitals or nursing homes and alert staff if a patient is wandering or falls [16,22,29].
- In smart home systems, various devices are controlled based on the location of the occupants, such as turning off lights in rooms that are not occupied or adjusting the temperature based on the presence of people in the room [8,9,10,12,14,15,19,21,22,23,24,25,26,30].
- In retail stores, to track the movement of customers and analyze their behavior, such as the areas where they spend the most time, the products with which they interact, and the path they take through the store.
- In robotics, to control the movement of robots and ensure their safety around humans.

Therefore, HWDD using wireless signals can enable various automated and intelligent systems to be more efficient, effective, and responsive to human needs and improve their well-being in terms of health and comfort [26].

Several studies have investigated the utilization of wireless signals for detecting human walking direction, speed, and location [9,10,31,32,33,34,35,36]. The previous works are based on RSSI and CSI amplitudes; however, they have serious limitations, such as degraded accuracy in small spaces, environmental changes, and a need for a specific device setup [9,10,31,34,36]. Moreover, these methods have relied only on a small subset of available signal streams from multi-antenna systems, resulting in limited predictive capabilities for determining the walking direction [34].

In this paper, we propose an approach that leverages machine and deep learning and all available signal streams of commercial multi-antenna Wi-Fi devices, enabling more accurate and reliable walking direction detection. We obtain the CSI values of signals transmitted via pairs of transmitting antennas and received via pairs of receiving antennas of 802.11n Wi-Fi devices. We use the Hampel filter algorithm and the discrete wavelet transform (DWT) to extract the features of the amplitude of the signal and the calibration to extract the features of the phase of the signal. The performance of four different classifiers, namely K-nearest neighbor (KNN), random forest (RF), the support vector machine (SVM), and the one-dimensional convolutional neural network (1D-CNN), was investigated using a pre-labeled dataset. Performance evaluations showed that SVM is an optimal classifier, achieving HWDD accuracy rates of 92.9%, 95.1%, and 89% for untrained data collected from classroom, meeting room, and both rooms, respectively. This high precision indicates that our approach effectively addresses the challenges of achieving high accuracy in HWDD in the presence of environmental changes and diversity in users. The major contributions of this paper are as follows:

- First, we thoroughly analyze the literature related to HAR in general and HWDD in particular.
- Then, we identify the major weaknesses of existing HWDD schemes.
- Next, we propose our new approach for HWDD, which uses the CSI of all available signal streams, the Hampel filter algorithm, and DWT to denoise and extract the features of amplitude, phase calibration, and finally, machine and deep learning algorithms to identify the human walking direction.
- Then, we perform extensive experiments under different conditions, such as various environments and diversity in users.
- Finally, we evaluate the performance of our approach using a variety of machine and deep learning classifiers, including RF, KNN, SVM, and 1D-CNN.

The remainder of this paper is organized as follows. Section 2 provides background information and presents a comprehensive review of the literature. Section 3 introduces a new approach to HWDD and describes the experiments and data collection methodologies. Section 4 presents the metrics and performance evaluation results. Finally, Section 5 concludes the article and discusses future work.

## 2. Background and Literature Review

### 2.1. Channel State Information

Channel state information (CSI), denoted as H(*f*,*t*) for carrier frequency *f* and time *t*, can be described using the channel frequency response (CFR). The relationship between the transmitted signal X(*f*,*t*) and the received signal Y(*f*,*t*) in the frequency domain is given by

(1) Y(f,t)=H(f,t)×X(f,t).

In Wi-Fi, a wireless channel consists of multiple orthogonal frequency division multiplexing (OFDM) subcarriers. For example, a 20 MHz wide Wi-Fi channel includes 56 subcarriers. Upon receiving a packet (a data unit in computer networks), the Wi-Fi receiver measures CSI for each subcarrier.

The 802.11n Wi-Fi devices have multiple antennas that enable them to send and receive packets using multiple signal streams: a stream per each transmit and receive antenna pair. The 802.11n receiver measures the CSI for each subcarrier and for each signal stream.

The CSI matrix is a representation of the channel information between the transmitting and receiving antennas in a wireless communication system. It provides vital information on the amplitude, phase, and frequency response of the channel, which are essential for efficient data transmission and reliable communication. The Wi-Fi receiver has the capability to generate a sampled version of the signal spectrum for each subcarrier, including complex numbers that represent both amplitude attenuation and phase shift. These data can be summarized as

(2) $$H_{i}=\left|\right|H_{i}\left|\right|e^{j∠H_{i}}$$

where $\left|\right|H_{i}\left|\right|$ represents the amplitude and $∠H_{i}$ represents the phase of the CSI at the *i*th subcarrier.

The dimensions of the CSI matrix are determined by the number of transmitting and receiving antennas, as well as the number of subcarriers within the channel bandwidth. Assuming one complex value per subcarrier, the size of the CSI matrix can be calculated as M×N×56 for a 20 MHz channel bandwidth and M×N×114 for a 40 MHz channel bandwidth. Here, *M* and *N* represent the number of transmitting and receiving antennas, respectively [37,38].

### 2.2. Literature Review

Currently, many HAR techniques exist that aim to track human gestures, activities, movements, counts, and walking directions using wireless signals in indoor environments. Some of them are summarized in Appendix A in Table A1, Table A2, Table A3 and Table A4. In the following subsections, we discuss the main contributions in each category.

#### 2.2.1. Human Gesture Detection

One of the potential areas where wireless signals can be employed is human gesture detection. There are many works in literature related to this area of study. For example, *Wisee* is the first wireless system that addressed the NLOS environment and can detect nine gestures with an accuracy of 94%. It used the analysis of Doppler shifts in wireless signals during transmission. However, the accuracy degraded with an increase in the number of interfering users [39]. In another work [40], the authors used CSI and deep learning techniques to recognize 12 gestures, and its accuracy was 98%. There is a similar work called *WiRoI*, but it used SVM classifiers instead of deep learning to recognize human movements. However, it was trained for only a single user [27]. In another similar work [41], the authors used CSI and KNN classifier to recognize five different hand gestures with an accuracy of 95% and 85% using amplitude and phase information, respectively.

The authors of *WiFinger* used CSI and multidimensional dynamic time wrapping to recognize eight finger gestures with an accuracy of 93% and 90% in LOS and NLOS conditions, respectively [30]. *FreeGesture* used CSI and a convolutional neural network (CNN) classifier to recognize six gestures with an accuracy of 95.8%. However, it was applied to only one user [21]. In [11], the authors used RSSI and one-dimensional CNN (1D-CNN) to recognize different hand gestures in the recognition area with an accuracy of 86.91%. *AirDraw* used CSI (phase and triangulation) to detect handwriting, with a median error of 2.2 cm [12]. *Wiga* used CSI and CNN–long short-term memory (LSTM) to detect yoga exercises with an accuracy of 97.7% and 85.6% for training and testing data, respectively [23]. *WiADG* used CSI and CNN classifiers to detect six gestures with an accuracy of 98% and 66.6% in the original environment and under environmental changes, respectively. However, noise removal was not used [24]. *FingerPass* used CSI and LSTM-based deep neural network (DNN) to detect finger gestures with an accuracy of 80% [25]. Table A1 in Appendix A includes a structured comparison of the works in this category.

#### 2.2.2. Human Activity Detection

We will now describe some of the works that aim to detect human activity using wireless signals. *PAWS* used RSSI and KNN classifiers to recognize six activities with an accuracy of 72.47% [42]. *WiFall* used CSI to detect falls with an accuracy of 90% and 94% using SVM and RF algorithms, respectively. However, it was designed and tested on a single user [13]. *E-eyes* used RSSI and CSI to recognize two activities by using clustering. They can accurately identify activities that are closely associated with specific locations, indicating a high correlation between activity and location. However, it was designed and tested on a single user [29]. *CARM* utilized CSI to recognize three activities by measuring the correlation between speed and movement and using a hidden Markov model (HMM) to identify specific activities with an accuracy rate of 96% [8]. *WiGest* used RSSI to recognize three different activities in NLOS scenarios with an accuracy of 87.5% for different users [43]. In [15], the authors used CSI and the class-estimated-basis space singular value decomposition non-negative matrix factorization (CSVD-NMF) for an activity recognition system and occupancy detection with an accuracy of 90.6% and 91%, respectively. *Freedetector* used CSI and RF classifiers to detect the presence of humans with an accuracy of 93.73%. However, it was designed and tested on a single user [16]. *DeepSense* used CSI and CNN to recognize different activities with an accuracy of 97.4% [26]. In [17], the authors used CSI and CNN–LSTM model to detect eight different activities with an accuracy of 96%. However, it was designed and tested on only one user, and due to the need for multiple receivers to achieve precise activity detection, their model was not considered to be cost-effective. In [18], the authors used CSI and CNN classifiers to detect five different activities with an accuracy of 78%. However, it was designed and tested on one user. *CDHAR* used CSI and the ensemble method to recognize five activities with an accuracy of 90% [19]. In [20], the authors used CSI and different machine learning classifiers to detect three activities with an accuracy of 95.6%. *FALAR* used CSI and CSVD-NMF to recognize six activities with an accuracy of 90% [22]. Some works in this category are summarized in Table A2 in Appendix A.

#### 2.2.3. Human Movement Detection

There are several studies pertaining to detecting human movement using wireless signals. For example, *WiWho* uses CSI and the decision tree classifier to detect human steps with an accuracy of 80% and 92% for six and two users, respectively [14]. *WiDMove* used CSI and SVM classifiers to identify the entrance and departure of users with an accuracy of about 80% [44]. *WifiU* analyzed the patterns of Wi-Fi signals in the frequency domain using SVM classifier to estimate the impact of the torso and legs on the Wi-Fi spectrum with an accuracy of 79.28%, 89.52%, and 93.05% for top-1, top-2, and top-3 based on 50 subjects, respectively. However, the experiment was carried out only in one place [45]. In [46], the authors used CSI and a linear discriminant classifier to count the number of people in different rooms with an accuracy of 74%. However, the accuracy decreased by about 20% in larger rooms. A more structured comparison of the abovementioned works can be found in Table A3 in Appendix A.

#### 2.2.4. Human Walking Direction Detection (HWDD)

HWDD is a topic of major interest in this paper. There are several works that use wireless signals for HWDD. For example, the authors of *WiDir* suggested the utilization of a Fresnel zone-based model to approximate the direction of a person’s movement by analyzing the phase shift in CSI, achieving a median error rate below 10 degrees. The system requires a specific device setup consisting of a minimum of three laptops to establish two Fresnel zones to operate accurately [9]. *WiDar* used CSI to estimate the velocity (speed and direction) of walking, as well as the location at a precision of a decimeter, analyzing Doppler frequency shifts. However, accuracy decreased as the user moved further away from the wireless link and the users were required to wear special clothing for the experiment [10].

*WiDet* used RSSI and CNN to detect human walking directions through corridors with an accuracy of 94.5% for a total of 163 walking events. However, RSSI is known to be susceptible to the shadowing effect and multipath, which can introduce errors and inaccuracies in signal measurements, and we did not find certain experiment details such as whether the experiment was conducted for a single user or multiple users, as well as the specific data training and testing size [31]. In [32], the authors used CSI amplitudes to detect the relationship between the extracted signals, the number of people, the speed of walking, and the direction of walking for 286 samples of three people with a precision greater than 90%. However, accuracy is degraded with different training sizes.

In [34], the authors proposed two techniques to determine the walking direction of a user passing through a corridor where special sensor nodes are installed on both sides at three different heights. The first technique involves an unsupervised technique that utilizes the variance of RSSI within short-term windows. This method determines the walking direction by analyzing the time delay between signal fluctuations in two selected streams. The second technique is a supervised approach that compares the measured signal in a chosen stream with a reference signal in the same stream using dynamic time warping (DTW). The accuracy of both methods ranges from 40% to 99%, depending on the chosen streams for 280 samples. However, the limitation of these techniques is their reliance on a small subset of streams to determine the direction of the walk, which may not provide the best performance. In [33], the authors used CSI to classify the direction and velocity of human walking with an accuracy rate of 91% for 136 samples from three people in four corridors and achieved a differentiation of human height with an accuracy of 76.7%. However, except for the height experiment, the collected data were limited to a single user.

*Gate-ID* used CSI amplitudes and deep learning techniques to detect human gait in two directions (left and right) with an accuracy of 90.7% [35]. *WiDIGR* used CSI based on the Fresnel zone and SVM classifier to detect walking direction with an accuracy of 78.28% and 92.83% for an apartment and empty rooms, respectively. However, the accuracy decreased by about 20% during testing. In addition, the accuracy degraded in small places and with changes in the environment and an increase in the number of people [36]. Interested readers can find a more structured comparison of the abovementioned works in Table A4.

## 3. System Design

**Figure 1:**
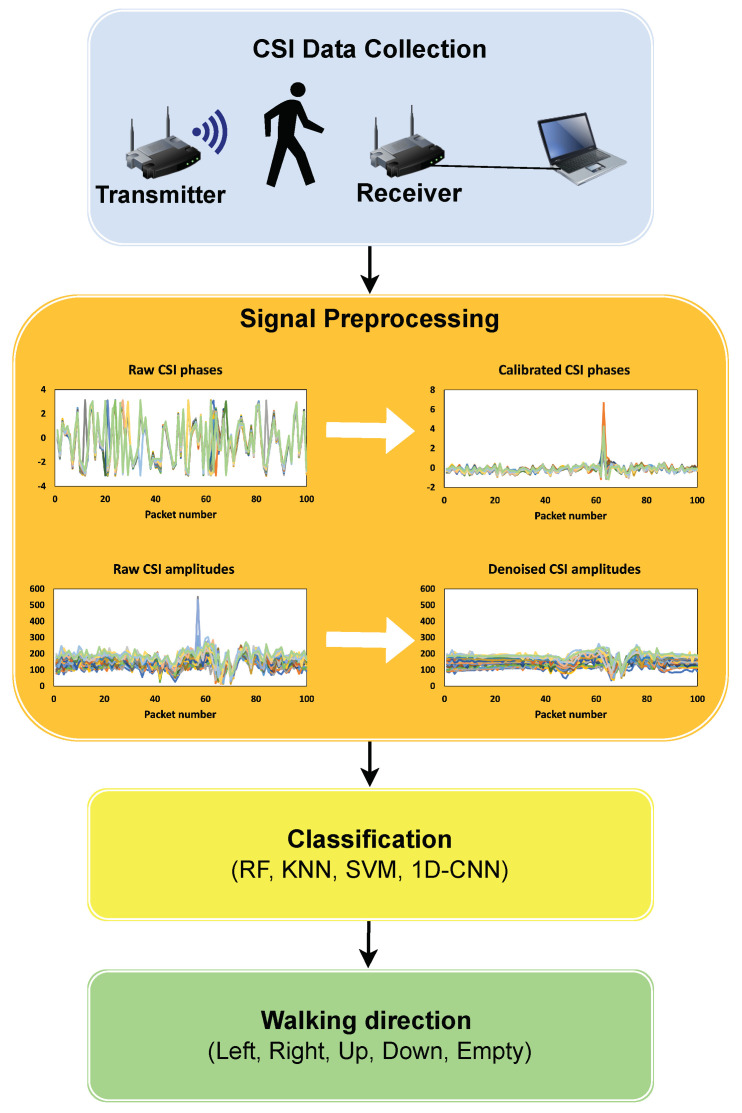
System overview.

In this paper, we propose a new approach for HWDD leveraging the CSI of Wi-Fi signals and machine and deep learning algorithms, as shown in Figure 1. Our system utilizes the CSI of all available signal streams, unlike the previous methods, where only a subset of the streams were used. Moreover, the proposed scheme relies on feature extraction techniques such as phase calibration, the Hampel filter algorithm, and DWT. In this section, we first discuss the setup of the experiment, the data collection method, data preprocessing, feature extraction, and walking direction recognition.

### 3.1. Experiment Setup

We explored three alternatives to obtain CSI data from Wi-Fi routers, namely the Linux 802.11n CSI Tool [38], Atheros CSI Tool [47], and Nexmon Channel State Information Extractor [48]. These tools require custom firmware and Linux wireless drivers, which require installation on devices instead of relying on vendor-provided solutions. Due to the obsolescence of the Network Interface Controller supported by the Linux 802.11n CSI Tool and the limited device compatibility of the Nexmon CSI Extractor, we used the Atheros CSI Tool. This open-source tool is designed for 802.11n measurement and experimentation, facilitating the extraction of comprehensive wireless communication details from Atheros Wi-Fi Network Interface Cards (NICs). This information includes CSI, received packet payload, data rate, timestamp, RSSI of each antenna, and other relevant metrics.

In our experiment, we used a laptop and a pair of TP-link TL-WDR4300 Wi-Fi routers with a channel bandwidth of 20 MHz and a frequency of 2.4 GHz (f or more details about the TP-Link TL-WDR4300 router, refer to the OpenWrt documentation: https://openwrt.org/toh/tp-link/tl-wdr4300_v1 (accessed on 1 September 2023)). These routers serve as a transmitter (TX) and receiver (RX), configured to operate in access point and client modes. The routers have two transmitting and two receiving antennas, resulting in a total of four signal streams, and the wireless communication details were extracted using 56 OFDM subcarriers as shown in Table 1. We installed OpenWrt on the routers, which is a Linux-based operating system designed for embedded devices. However, we used custom OpenWrt, which is modified to extract the CSI data to the user space [47].

In our experiments, one of the Wi-Fi routers was configured as a transmitter and the other as a receiver. The transmitter periodically (every 50 ms) sends a dummy packet to a receiver. Each dummy packet is transmitted through two antennas at the transmitter. In other words, two copies of the same dummy packet are transmitted at the transmitter, a copy per antenna. The receiver uses two of its antennas to receive. When a receiver receives the dummy packet, it actually receives four copies of it. The first two copies are received at one antenna; these copies are transmitted through different antennas at the transmitter. The second two copies are received at the second antenna.

The receiver obtains the CSI vector for each received copy of the packet. Since these copies are transmitted through two different transmit antennas and received at two different receive antennas, the surrounding environment, including human activity in the vicinity, affects the signals of the packets differently, and thus, the four vectors are not identical. The four vectors constitute a CSI matrix together. The receiver then sends the CSI matrix and its associated receipt timestamp to the laptop through a wired Ethernet link. The laptop is then responsible for handling, storing, and visualizing the computed CSI data.

### 3.2. Data Collection

Each vector in the CSI matrix has 56 entries, an entry per OFDM subcarrier. Each entry in the CSI vector is called a CSI value. Each CSI value includes two items: an amplitude and the phase of the subcarrier. Thus, for each packet, the laptop records of 56×4×2=448 CSI values.

In the experiments, the user performs certain activities for five seconds. For example, the user moves from left to right. During this time, the transmitter is configured to send 100 packets, that is, at a rate of 20 packets/s or at 50 ms intervals, so user activity affects the CSI matrices of the packets received at the receiver. A hundred CSI matrixes together constitute one *sample*.

**Figure 2:**
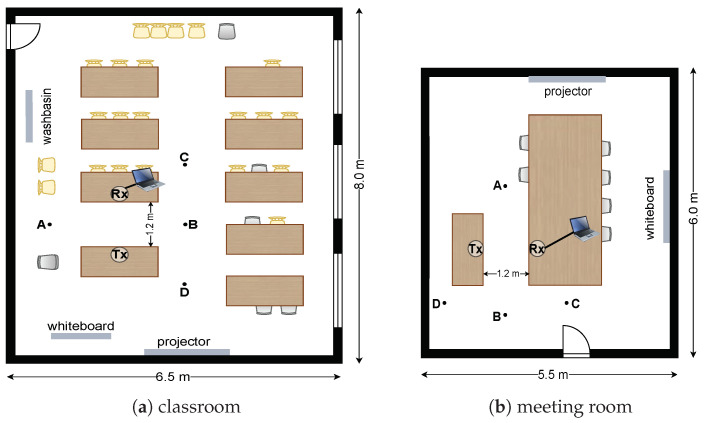
Two indoor environments.

Figure 2 depicts the two indoor environments, namely a classroom (6.5 m × 8.0 m) and a meeting room (5.5 m × 6.0 m), used for experiments and four points, between which the users walked during experiments. There are more tables and chairs in the classroom compared to the meeting room. The position of objects can affect the propagation of the signal in different ways, leading to different multipath effects in different environments. We set the transmitter and receiver at a height of 0.75 m in each environment, with a distance of 1.2 m between them.

The samples were collected for four different activities, i.e., walking directions: left (from A to B), right (from B to A), up (from C to D), and down (from D to C). Nine volunteers with different genders (seven males and two females), ages (from 17 to 29 years old), heights (from 1.68 m to 1.88 m), and walking styles participated in the experiments. Each volunteer walked in each direction about five to seven times in both rooms, resulting in 186 and 182 samples from the classroom and meeting room, respectively. For example, if each of the nine volunteers had walked in each of the four directions five times, the total number of samples collected would be 5 × 4 × 9 = 180 samples for one room.

### 3.3. Data Preprocessing

#### 3.3.1. Phase Calibration

**Figure 3:**
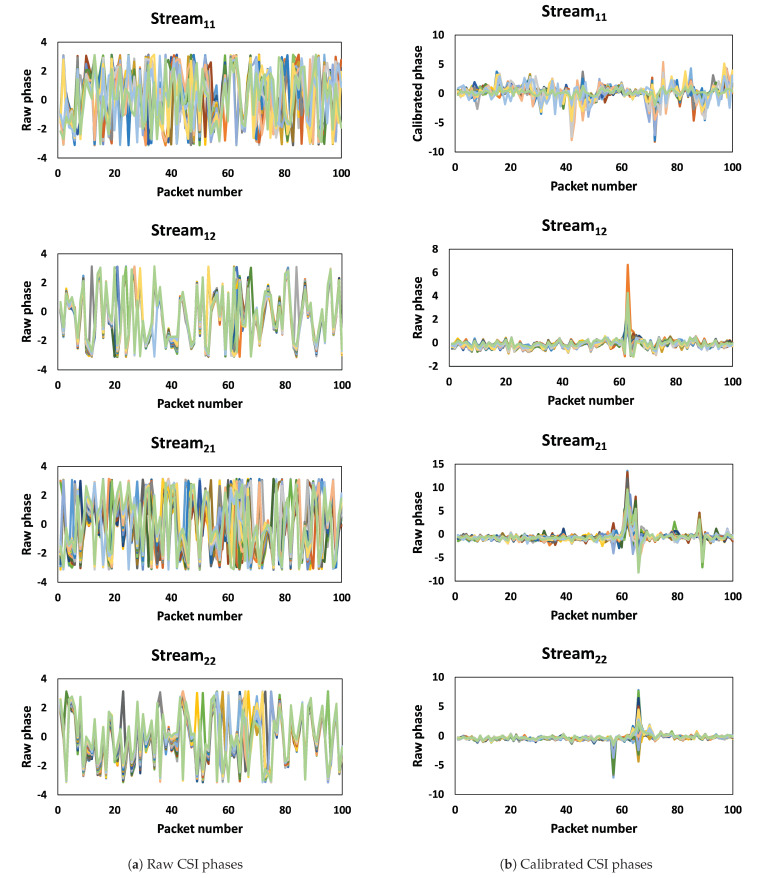
Raw and calibrated phases for 30 subcarriers.

**Figure 4:**
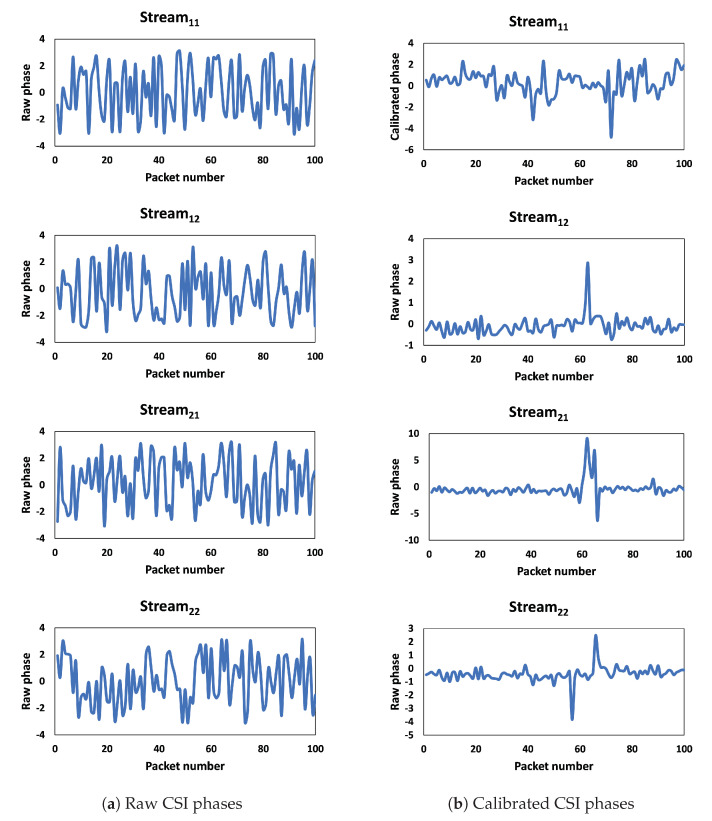
Raw and calibrated phase for one subcarrier.

Figure 3 depicts the phases of one sample for the first thirty subcarriers. The stream_ij_ represents CSIs of the packet copies that are transmitted from TX antenna *i* and received at RX antenna *j*. The raw phase information becomes impractical for usage due to the impact caused by the carrier frequency offset (CFO) and sampling frequency offset (SFO). CFO arises from a lack of synchronization in timing and phases between the transmitter and receiver before transmitting the packet [49]. Figure 3a and Figure 4a depict the raw phases of the first thirty subcarriers and a single subcarrier of one sample, respectively. It is obvious that the raw phase is not sufficient to provide insights into walking activity. However, Figure 3b and Figure 4b show that after calibration, the new phase data exhibits reduced noise, and the change in phase during walking activity is noticeable.

#### 3.3.2. Denoising the CSI Amplitudes Using the Hampel Filter Algorithm

Amplitudes are affected by various types of outlier and noise, from limited bandwidth to transition rate and power adaptations, as well as thermal noise. Consequently, signal outliers arise that are not attributable to human actions. To address this problem, the Hampel filter algorithm [50] is applied. This algorithm applies the median and median absolute deviation to identify the outliers’ location. The Hampel filter is a robust method for outlier detection that uses a sliding window approach to identify and remove data points that deviate significantly from the median value in that window.

#### 3.3.3. Feature Extraction from CSI Amplitudes Using Discrete Wavelet Transform

The wavelet transform is a powerful technique that allows for the precise localization of events in both time and frequency domains. By utilizing the wavelet transform as a feature extractor, we can achieve higher accuracy in event detection compared to statistical features. The approach is based on the idea of analyzing time series data on multiple scales. With smaller-scale wavelets, the transformed signal will exhibit large peaks during the event, allowing the identification of the occurrence of the event in the time domain [31].

**Figure 5:**
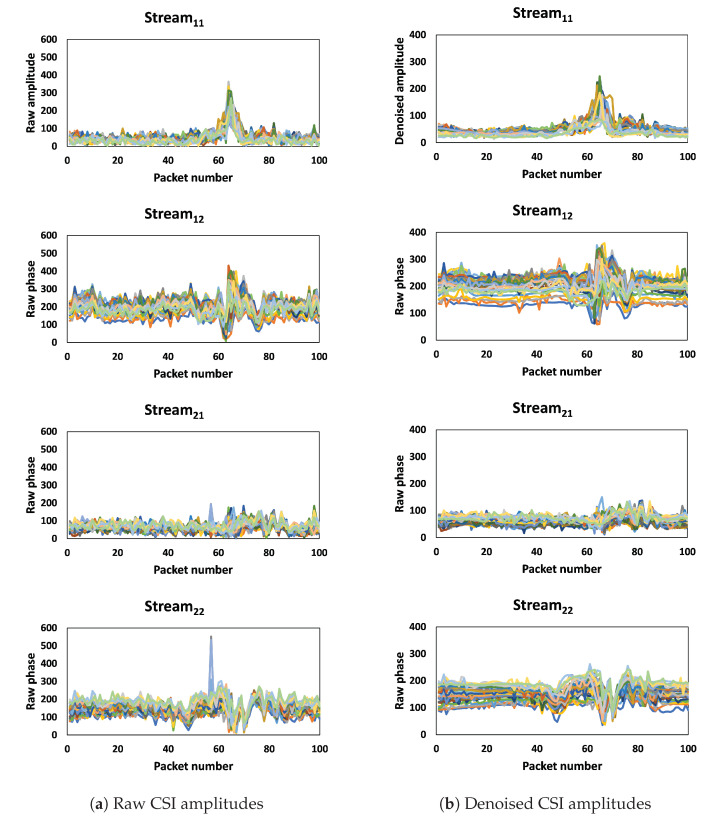
Outlier removal and noise reduction based on Hampel filter algorithm and DWT for 30 subcarriers. Different colors represent different subcarriers.

**Figure 6:**
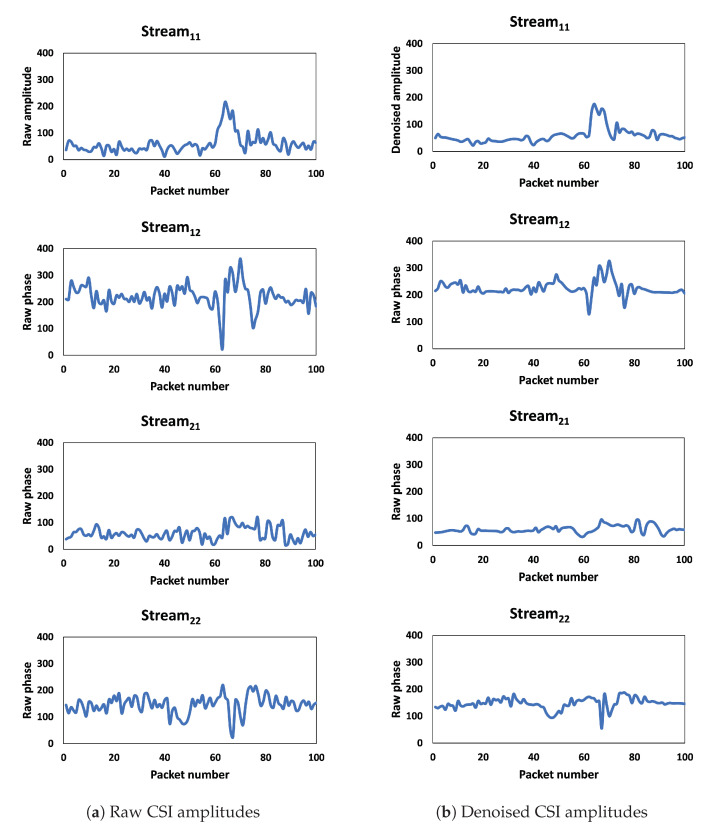
Outlier removal and noise reduction based on Hampel filter algorithm and DWT for one subcarrier.

To extract the useful features of CSI amplitudes, we used DWT. The DWT algorithm can be used to decompose CSI into multiple levels of wavelet coefficients, each representing different frequency bands in the signal. The high-frequency wavelet coefficients represent the noise and other high-frequency components of the signal, while the low-frequency coefficients represent the smoother components of the signal. By filtering out the high-frequency coefficients representing noise and high impulses, as shown in Figure 5a and Figure 6a, and retaining the low-frequency coefficients, the denoised signal is obtained in Figure 5b and Figure 6b.

#### 3.3.4. Comparison of Denoised Phase and Amplitudes for Different Activities

**Figure 7:**
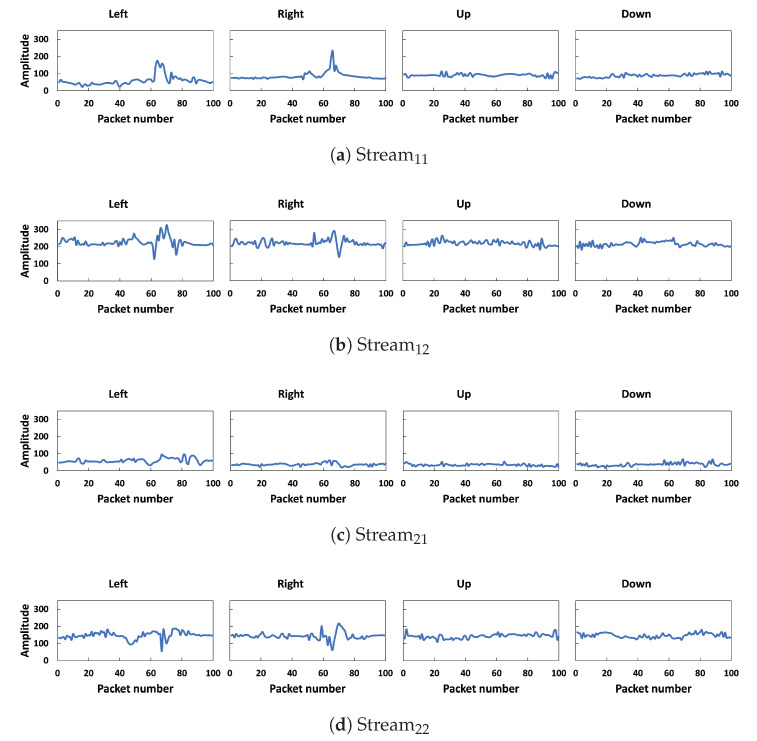
Denoised amplitudes of four walking directions for one subcarrier.

**Figure 8:**
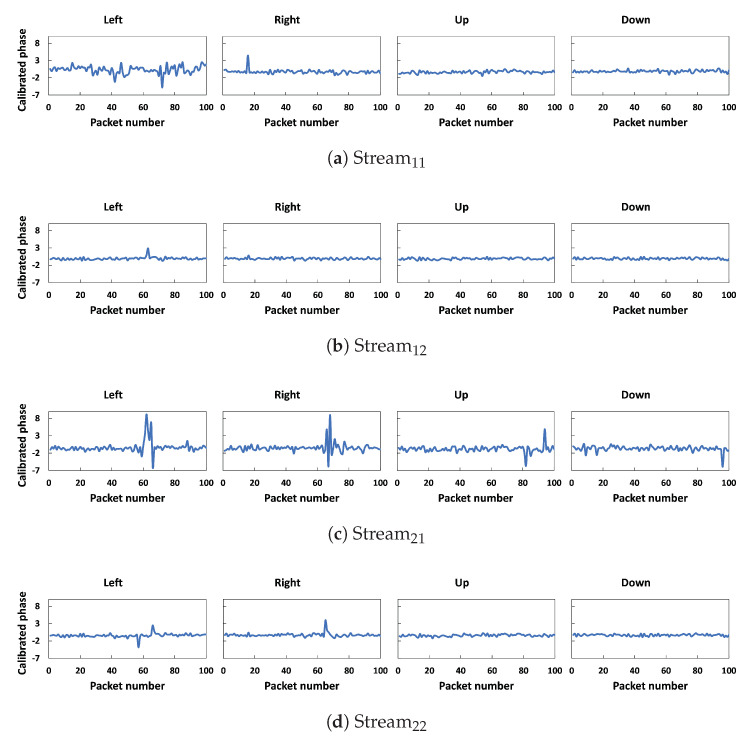
Calibrated phases of four different walking directions for one subcarrier.

We removed the noise from CSI phases using calibration and the outliers and noise from amplitudes using the Hampel filter algorithm, selected the important features, and removed the remaining noise using DWT. Figure 7 and Figure 8 show the comparison of denoised amplitudes and phases, respectively, for different walking directions. For *left* and *right* walking directions, both amplitudes and phases have sharp fluctuations for all streams, which indicates the moment when the user crosses the LOS line between the transmitter and the receiver. Moreover, the data presented in the figures demonstrate a noticeable difference in the amplitude and phase patterns produced through different activities. Our observations have led us to believe that CSI data can be utilized to recognize different walking directions.

The CSI for the received packet consists of $N_{tx}\times N_{rx}\times N_{s}$ matrix, where $N_{tx}$ and $N_{rx}$ denote the antenna numbers for the transmitter and receiver, respectively, and $N_{s}$ represents the subcarriers across the OFDM channel. Specifically, with $N_{tx}$ = 2 and $N_{rx}$ = 2, one packet consists of four distinct CSI values or streams, each containing values for 56 subcarriers. We selected only four subcarriers out of the total 56, which significantly reduces the computational complexity. This reduction enables faster and more real-time direction detection in various practical scenarios. We specifically chose subcarriers from different ranges within the 56 subcarriers to ensure that they represent frequencies that are far from each other and react differently to the changes caused by human walking. These data can be represented in the following format:

$CSI^{1}=\left\{CSI^{1,11},CSI^{1,22},CSI^{1,33},CSI^{1,44}\right\}$,
$CSI^{2}=\left\{CSI^{2,11},CSI^{2,22},CSI^{2,33},CSI^{1,44}\right\}$,
$CSI^{3}=\left\{CSI^{3,11},CSI^{3,22},CSI^{3,33},CSI^{1,44}\right\}$,
$CSI^{4}=\left\{CSI^{4,11},CSI^{4,22},CSI^{4,33},CSI^{1,44}\right\},$

where $CSI^{i,j}$ is the 〈amplitude, phase〉 for i-th stream and j-th subcarrier.

### 3.4. Activity Recognition

In this work, we use several machine learning and deep learning techniques to classify the directions of human walking. In particular, we employed four popular classifiers, namely SVM, RF, KNN, and 1D-CNN. SVM is a supervised learning model that identifies patterns in data. To handle non-linear classification problems, input samples are mapped to a high-dimensional feature space using a kernel function, and a maximum-margin hyperplane is then identified in this space.

RF, on the other hand, is an ensemble learning method that enhances classification performance through the fusion of multiple decision trees. Each tree is constructed using a random subset of the training data, and the final classification decision is made based on the majority vote of the trees.

KNN, a simple but effective classification method, relies on measuring the distance between feature values. The closest point in a scale space is then used to classify the new data point. The distance can be computed using various metrics, such as the Euclidean or Manhattan method.

Finally, 1D-CNN is a deep learning model specifically designed for one-dimensional data such as time series or sequences. The 1D-CNN leverages convolutional layers to automatically learn hierarchical representations from the input data, capturing local patterns that are essential for the direction of human walking in our work.

Our dataset is organized as follows. As mentioned before, we collected in total 186 + 182 = 368 samples of four walking directions from nine users in two rooms. In addition, we also recorded 20 samples for the case of no activity, i.e., no people. Therefore, we have 206 samples from the classroom and 202 samples from the meeting room, totaling 408 samples when combined (classroom + meeting room). Moreover, samples are reorganized as follows. As we discussed earlier, each sample includes 100 CSI matrices, i.e., one CSI matrix per packet. An original CSI matrix includes 56×4=224 CSI values; however, we further use CSIs of only four subcarriers, and thus now, each matrix includes 4×4=16 CSI values.

To train and test our model, we randomly partitioned the dataset: 80% for data training and the remaining 20% for testing. This approach was used to ensure that the model was tested on new data. We used 10-fold cross-validation to determine the best hyperparameters for each algorithm. Specifically, we assessed the performance of RF with a depth of 12, SVM with a linear kernel, and KNN with three neighbors.

**Figure 9:**
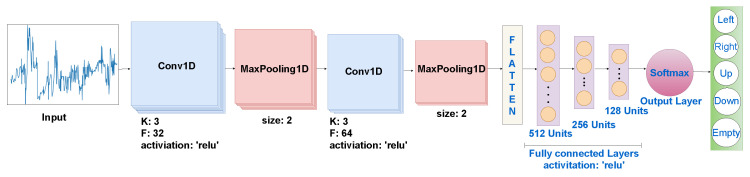
One-dimensional CNN architecture.

The 1D-CNN model consists of two convolutional layers with rectified linear unit (ReLU) activation functions, followed by max-pooling layers to capture and emphasize key features. The flattened output is then connected to three dense layers (of sizes 512, 256, and 128), each activated via ReLU. The final layer utilizes a softmax activation function to produce probabilistic predictions across five different classes corresponding to distinct human walking directions, as shown in Figure 9. The model is trained using the Adam optimizer with a sparse categorical cross entropy loss function over 100 epochs.

## 4. Performance Evaluation

### 4.1. Metrics

After creating and training the model, it is necessary to evaluate how well the model performs. For this purpose, it is necessary to use a test dataset that is different from the data on which the model is trained. The predicted values are then compared with the actual values. For different types of deep learning and machine learning problems, different quality assessment tools need to be used. For classification tasks, for example, the following tools can be used: error matrix, precision, recall, f1 score, and so on. Such tools are called metrics and are selected depending on the specifics of the task [51]. To assess the effectiveness of our system, we use several evaluation metrics: confusion matrix, recognition accuracy, precision, recall, and f1 score. The confusion matrix displays the classification results for each walking direction and the corresponding actual walking direction performed by the user. Each cell within the matrix denotes the proportion or percentage of accurately classified activities. However, the recognition accuracy represents the percentage of human walking directions accurately classified by our system. Precision quantifies the ratio of correctly identified positive instances to the overall number of positive classifications generated by the model. In contrast, recall represents the ratio of correctly identified positive instances to the total number of positive instances present in the dataset. The f1 score serves as the harmonic mean of precision and recall, offering a unified metric that strikes a balance between both evaluation measures. Together, these metrics provide a comprehensive evaluation of the performance of our system.

(3) $$\mathrm{Accuracy}=\frac{\mathrm{TP}+\mathrm{TN}}{\mathrm{TP}\;+\;\mathrm{TN}\;+\;\mathrm{FP}\;+\;\mathrm{FN}}\times 100\%$$

(4) $$\mathrm{precision}=\frac{\mathrm{TP}}{\mathrm{TP}\;+\;\mathrm{FP}}$$

(5) $$\mathrm{recall}=\frac{\mathrm{TP}}{\mathrm{TP}\;+\;\mathrm{FN}}$$

(6) $$f1-\mathrm{score}=2\times \frac{\mathrm{Precision}\times \mathrm{Recall}}{\mathrm{Precision}+\mathrm{Recall}}$$

where TP represents true positives, TN represents true negatives, FP represents false positives, and FN represents false negatives.

The source code for preprocessing, training, and testing is published on Github [52]. A raw dataset that we gathered for this study is available on Figshare [53].

### 4.2. Impact of Environment Change

**Figure 10:**
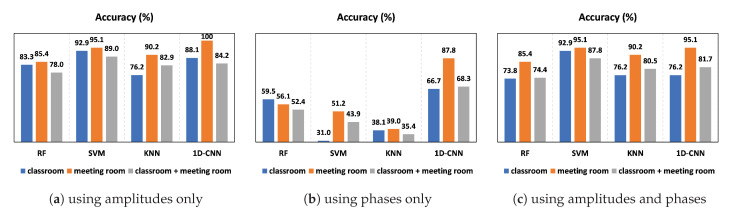
System accuracy.

Figure 10 depicts the comparison of the HWDD accuracy performance of different classifiers. Figure 10a shows the comparison for the case when only CSI amplitudes are used. When trained and tested on combined data from both rooms, the RF, SVM, KNN, and 1D-CNN classifiers have accuracy rates of 78%, 89%, 82.9%, and 84.2%, respectively. In general, SVM is the most consistent and performing model in both environments. Similarly, SVM achieved high accuracy rates of 92.9% and 95.1% in the case of classroom and meeting room data, respectively. This difference in performance is caused by variations in the data collected from each environment. The classroom has more tables and chairs that can reflect signals, making it more challenging to accurately classify the walking direction. In contrast, a meeting room has less furniture and is smaller, allowing the model to perform better.

Figure 10c shows the comparison for the case where classifiers are trained and tested with both CSI amplitudes and phases. For both datasets in both rooms combined, SVM performed consistently well compared to RF, KNN, and 1D-CNN, achieving a high accuracy rate of 87.8%. For the classroom and meeting room, SVM achieved accuracies of 92.9% and 95.1%, respectively. However, when we used only CSI phases for data training and testing, the accuracy results were not comparable to the CSI amplitudes. The one-dimensional CNN achieved the highest accuracy of 66.7%, 87.8%, and 68.3% for the classroom, meeting room, and both rooms, respectively, as shown in Figure 10b.

Overall, the results of SVM are promising for HWDD using CSI amplitudes. We explore the use of CSI amplitudes, CSI phases, and a combination of both in our approach. Our analysis revealed that the CSI amplitudes exhibited the most consistent performance in the face of environmental variations, different users, and classifiers, emphasizing their superiority over other techniques.

Table 2 displays the confusion matrices of the SVM classifier, showcasing its performance in different environments using CSI amplitudes. In the classroom, our approach achieved an average accuracy of 92.9%. It accurately recognized the *right* and *up* directions with rates of 88.89% and 77.78%, respectively. Furthermore, it correctly detected all other activities with an accuracy rate of 100%. In the meeting room, our approach achieved an average accuracy of 95.1%. It successfully recognized the *left* direction with an accuracy rate of 85.71%, and the *down* and *up* directions with an accuracy rate over 75%. Similarly, it accurately classified all other activities with an accuracy rate of 100%. Finally, in both rooms, our approach achieved an average accuracy of 89%. It accurately recognized the *down*, *left*, *right*, and *up* directions with an accuracy rate greater than 84%. The *empty* activity was also identified correctly, with an accuracy rate of 100%.

**Figure 11:**
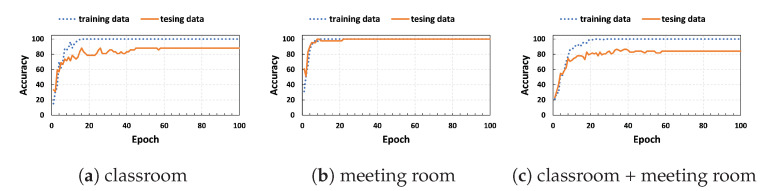
System accuracy of 1D-CNN classifier using CSI amplitudes.

In Figure 11, the accuracy of the 1D-CNN classifier is presented for training and testing data using CSI amplitudes over 100 epochs. In the classroom, our model achieved accuracy rates of 100% and 88.1% for training and testing data, respectively, as shown in Figure 11a. In the meeting room, the 1D-CNN classifier performed well compared to RF, SVM, and KNN, achieving high accuracy rates of 100% for both training and testing data, as illustrated in Figure 11b. For the combined data from both rooms, our model achieved accuracy rates of 100% and 84.2% for training and testing data, respectively, as depicted in Figure 11c. Overall, the model performed well in small spaces. However, its performance in large and diverse rooms was not as consistent as that of the SVM classifier. To enhance the reliability and robustness of deep learning algorithms, it is suggested to collect more samples.

Table 3 shows the comparison of the precision, recall, and f1 score for different environments. The SVM achieved the best performance using CSI amplitudes. The results showed that the system achieved high precision and a f1 score greater than 80% for all activities. This indicates that the system has a high level of accuracy in identifying each activity, with an extremely low rate of false positives. The system also achieved a recall rate greater than 85% for all activities, except for the *up* activity, which had a recall rate of 78%. These results suggest that the system can accurately detect and classify human activities with a high level of consistency.

### 4.3. Impact of Individual Diversity

**Figure 12:**
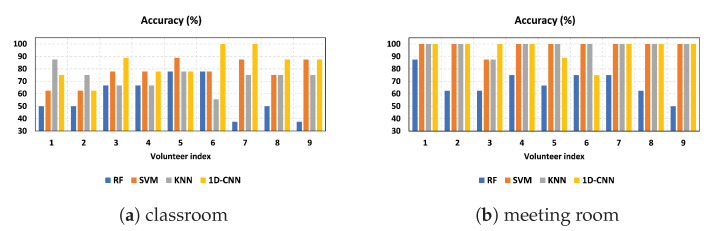
Accuracy for increasing the number of individuals in two indoor environments using CSI amplitudes.

Figure 12 depicts the accuracy performance for an increasing number of individuals. To evaluate the consistency of our approach among diverse users, we enlisted the participation of nine volunteers in the experiment. The selected volunteers have different ages, heights, weights, and genders. Our proposed method utilizing CSI amplitudes yielded exceptional results in the meeting room, as depicted in Figure 12b. SVM and KNN achieved 100% accuracy across all cases except for one where the accuracy rate was 87.5%. Additionally, 1D-CNN also produced the highest possible accuracy rates in most cases except for two cases where accuracy was 88.9% and 75%, respectively.

According to Figure 12a, in the classroom, the 1D-CNN achieved an average accuracy of over 84%, while the SVM and KNN classifiers achieved average accuracy rates of over 77% and 72%, respectively. In contrast, the accuracy of the RF is not comparable to that of other classifiers for both environments. These findings highlight the importance of using 1D-CNN and SVM classifiers with CSI amplitudes to improve accuracy, especially for different users and environments. One-dimensional CNN and SVM outperformed RF and KNN when training each user individually because they are skilled at picking up detailed patterns from CSI amplitudes. This allows them to create more accurate models for each user’s unique wireless signal variations, resulting in better performance in distinguishing activities.

### 4.4. Impact of Increasing the Number of People

**Figure 13:**
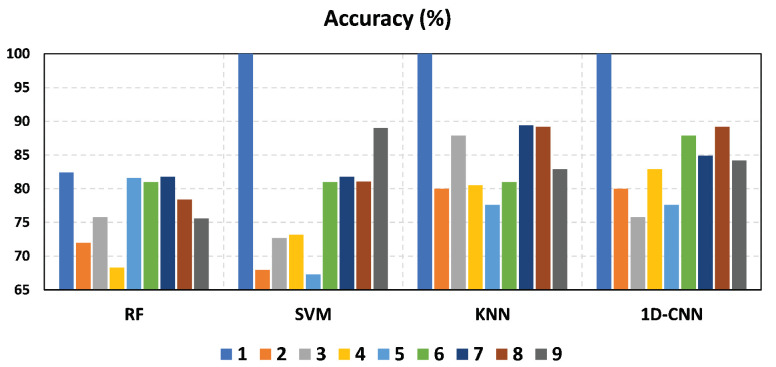
Accuracy of increasing the number of people in two indoor environments using amplitudes.

The findings of this study, as illustrated in Figure 13, indicate that our proposed method, which utilizes CSI amplitudes and the SVM classifier, yields high accuracy in both environments as the number of participants increases from one to nine. We collected data from both rooms for each participant, where the number “1” refers to the data of both rooms for one user, “2” represents the data of both rooms for two users, and so on. As the number of participants increases, the accuracy of the SVM classifier also increases. On the contrary, the accuracy of the RF, KNN, and 1D-CNN classifiers deteriorates.

### 4.5. Impact of Training Size

**Figure 14:**
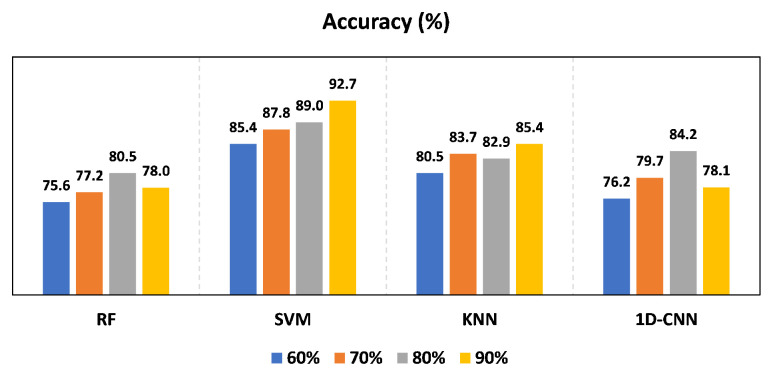
Impact of different training size using amplitudes.

Our proposed method has demonstrated the ability to achieve comparable results using a limited number of training samples, making it a more practical option for real-world applications. Using the CSI amplitudes and the SVM classifier in the combined data of both rooms, our approach was able to achieve high accuracy, even with varying sizes of training data for both environments, as depicted in Figure 14. The SVM classifier yielded similar results, with slight differences observed when different training sizes were used. SVM consistently performs well with varying amounts of training data due to its capability to construct an optimal hyperplane for classification. This is especially effective in high-dimensional spaces like those formed from CSI amplitudes, contributing to its stability across diverse training set sizes. These results indicate the robustness of our method across different data testing sizes. To further improve the performance and effectiveness of our approach, we can collect more samples to increase the size of the data training in the future.

### 4.6. Robustness

The results of the experiment demonstrate that the accuracy remains consistent with the increasing number of volunteers when using the SVM classifier and the CSI amplitudes in both rooms in Figure 13. Furthermore, when using different training sizes, the accuracy remained comparable in both rooms, according to Figure 14. The recognition accuracy achieved was 92.9%, 95.1%, and 89% in the classroom, meeting room, and both classroom and meeting room, respectively, as shown in Figure 10a. These results demonstrate that our system is robust to different individuals, environments, group sizes, and training sizes.

**Figure 15:**
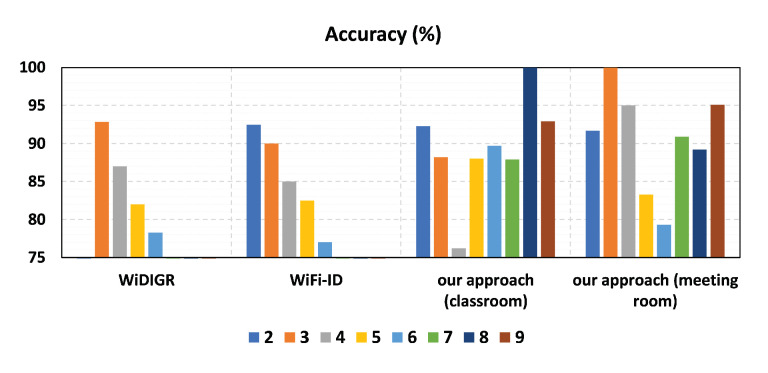
Comparison with the baseline recognition systems.

The performance of our approach by utilizing CSI amplitudes and SVM classifier in two indoor environments was evaluated and compared with two other indoor recognition systems, namely WiFi-ID [54] and WiDIGR [43], in two common indoor scenarios. Figure 15 shows the performance of each system, from the smallest to the largest group size of volunteers. The results indicate that the average accuracy of all systems decreases as the group size increases. In particular, our approach outperformed the other two systems, which rely solely on feature extraction and cannot accurately reflect movement information. Therefore, the reason our approach performed significantly better than the other systems is that those systems were not able to obtain precise movement information.

## 5. Conclusions and Future Work

In this paper, we introduced a device-free method that can precisely identify the direction of human walk using the CSI of Wi-Fi signals and machine and deep learning algorithms. Raw CSI signals are first calibrated and effectively denoised using Hampel filter and DWT algorithms. We conducted extensive experiments in two indoor environments. Our system, using SVM and CSI amplitudes, achieved recognition accuracy rates of 92.9%, 95.1%, and 89% in the classroom, meeting room, and both rooms, respectively. Additionally, our system, employing 1D-CNN and CSI amplitudes, demonstrated recognition accuracy rates of 88.1%, 100%, and 84.2% in the classroom, meeting room, and both rooms, respectively.

Our experiments consistently proved the robustness of our system in various scenarios. The accuracy remained stable even with an increasing number of volunteers and different training sizes, different individuals, environments, group sizes, and training data variations. Our approach demonstrates versatility in its applicability in various scenarios. It can be effectively employed to track the walking directions of customers in retail stores. Furthermore, in security systems, it proves valuable for monitoring human walking directions and detecting unauthorized access in restricted areas. However, our study has limitations, including a small dataset that may impact the accuracy of deep learning algorithms. Volunteers walking in straight lines limited the diversity of observed environmental conditions, although this assumption does not significantly impact system usability in common settings like homes or offices, where straight walkways are prevalent. Additionally, the study lacks samples collected in the presence of multiple individuals.

Our future research work will focus on improving the accuracy of human walking direction detection in the presence of multiple individuals and in the collection of more samples. We aim to enhance the efficiency and accuracy of activity recognition in complex and dynamic indoor environments. 

## Figures and Tables

**Table 1:** Collected dataset characteristic.

Activities	Left, Right, Up, Down, Empty
# of participants involved	9
# of environments used	2
Wi-Fi router	TP-link TLWDR4300
Channel bandwidth	20 MHz
Frequency	2.4 GHz
Antennas	2TX × 2RX
# of subcarriers	56

**Table 2:** Confusion matrix of SVM classifier using CSI amplitudes.

Activity	Classroom					Meeting Room					Classroom + Meeting Room				
	Empty	Down	Left	Right	Up	Empty	Down	Left	Right	Up	Empty	Down	Left	Right	Up
Empty	100	0	0	0	0	100	0	0	0	0	100	0	0	0	0
Down	0	100	0	0	0	0	92.86	0	0	7.14	0	86.36	0	4.55	9.09
Left	0	0	100	0	0	0	0	85.71	0	14.29	0	5.88	88.24	0	5.88
Right	0	0	0	88.89	11.11	0	0	0	100	0	0	0	7.69	84.62	7.69
Up	0	22.22	0	0	77.78	0	0	0	0	100	0	100	0	0	90

**Table 3:** Comparison between different metrics and environments using CSI amplitudes.

Activity	Classroom			Meeting Room			Classroom + Meeting Room		
	Precision	Recall	f1 Score	Precision	Recall	f1 Score	Precision	Recall	f1 Score
Empty	1.0	1.0	1.0	1.0	1.0	1.0	1.0	1.0	1.0
Down	0.83	1.0	0.91	1.0	0.93	0.96	0.86	0.86	0.86
Left	1.0	1.0	1.0	1.0	0.86	0.92	0.94	0.88	0.91
Right	1.0	0.89	0.94	1.0	1.0	1.0	0.92	0.85	0.88
Up	0.88	0.78	0.82	0.80	1.0	0.89	0.82	0.90	0.86

## Data Availability

We provide an open dataset that we gathered during our study on Figshare [53].

## Abbreviations

The following abbreviations are used in this paper:

HAR	Human activity recognition
LOS	Line-of-sight
NLOS	Non-LOS
RSSI	Received signal strength indicator
CSI	Channel state information
HWDD	Human walking direction detection
DWT	Discrete wavelet transform
RF	Random forest
SVM	Support vector machine
KNN	K-nearest neighbor
CFR	Channel frequency response
OFDM	Orthogonal frequency division multiplexing
CNN	Convolutional neural network
1D-CNN	One-dimensional CNN
LSTM	Long short-term memory
DNN	Deep neural network
CSVD-NMF	Class-estimated-basis space singular value decomposition non-negative matrix factorization
	HMM	Hidden Markov model
DTW	Dynamic time warping
CFO	Carrier frequency offset
SFO	Sampling frequency offset

## Appendix A

**Table A1:** Comparison between Wi-Fi-based system of human gesture detection.

System	Objective of Sensing	Methodology	Performance	Complexity	Devices
WiSee [39]	gestures: strike, drag, kick, bowling, punch, circle, push, dodge	Doppler shift, pattern matching	94%	no training needed	USRP-N210 hardware
Deep transfer learning for gesture recognition with Wi-Fi signals [40]	draw X, swipe right, punch 2 times, drag, circle, bowling, push, pull, swipe left, draw tick, enlargement, and shrink	CSI-based, classification with CNN–SVM, FT-CNN	97% using FT CNN and 95.17% using CNN-SVM	training needed	TP-Link router laptop with Intel 5300 Wi-Fi network interface card (NIC)
WiRoI [27]	gestures: punch out, push forward, swipe left, cut down	CSI-based, classification with SVM	88%	training needed	two mini-PCs were equipped with Ubuntu operating systems running on the Linux 4.2.0 kernel
Dynamic gesture recognition using wireless signals with less disturbance [41]	five hand gestures	CSI-based, classification with KNN, ICA to eliminate disturbance, to reduce data dimensionality and eliminate noise	the amplitude and phase information, respectively, yielded accuracy rates of 95% and 85%	training needed	two computers were set up, each one equipped with three-antenna Intel 5300 wireless network cards
Wi-Finger [30]	finger gestures: flip up/down, swipe left/right, circle left/right, and zoom in/out	CSI-based, classification with multi-dimensional dynamic time wrapping	93%	training needed	in an 802.11n Wi-Fi network, a Dell LATITUDE E5540 Laptop was used as a single Wi-Fi device, while a LINKSYS E2500 N600 Wireless Router served as the wireless Access Point
FreeGesture [21]	gestures: down, up, pull, push, left, right.	CSI-based, classification with CNN	95.8%	training needed	two TP-LINK N750 routers
Dynamic Hand Gesture Detection and Recognition with Wi-Fi Signal Based on 1D-CNN [11]	hand gestures: (7 characters “ABCDEFG”)	RSSI-based, classification with 1D-CNN	86.91%	training needed	the transmitter utilized in this setup is a widely used IoT Wi-Fi device called CC3200, while the receivers consist of two Universal Software Radio Peripherals (USRPs)
AirDraw [12]	handwriting detection	CSI-based, phase tracking and triangulation	median error lower than 2.2 cm	no training needed	three mini-PCs were utilized, with one serving as the transmitter (TX) and the other two as receivers (RXs). All devices employed Ubuntu 14.04 LTS and were equipped with Intel 5300 Wi-Fi NICs
Wiga [23]	yoga detection	CSI-based, CNN-LSTM	85.6%	training needed	two desktop computers were equipped with Atheros 9590 802.11n Wi-Fi NICs and operated on the Ubuntu 10.04 operating system
WiADG [24]	gestures: (left, right, roll left, roll right, pull, and push)	CSI-based, classification using CNN	98% in the original environment and 66.6% under environment changes	training needed	two TP-LINK N750 wireless routers, with one serving as the transmitter (TX) and the other as the receiver (RX)
FingerPass [25]	finger gestures (wave up, wave down, wave left, wave right, zoom out, and zoom in, circle left, circle right)	CSI-based, classification using LSTM-based DNN	80%	training needed	a laptop used in the experiment was an HP Pavilion 14, featuring an Intel Wi-Fi Link 5300 network interface card (NIC); this configuration allowed for the provision of 30 subcarriers on the CSI of Wi-Fi signals

**Table A2:** Comparison between Wi-Fi-based system of human activity detection.

System	Objective of Sensing	Methodology	Performance	Complexity	Devices
WiFall [13]	Activity: falling	CSI-based, one-class SVM model was employed, utilizing a set of seven features	The detection accuracy achieved was 87% with a false alarm rate of 18%	training needed	the Intel Wi-Fi Link 5300, an 802.11n network interface card (NIC)
PAWS [42]	Walking, standing, sitting, running, sleeping, falling, empty	RSSI-based, fusion algorithm with two features	97.47%	training needed	COTS Wi-Fi routers
E-Eyes [29]	Daily activity: washing dishes, talking on the phone	CSI-based, classification of in-place and walking activity	96%	clustering needed	the laptops utilized in the experiment were equipped with Intel 5300 NIC cards
CARM [8]	Sitting down, walking, falling	CSI-based, CSI speed model, and features derived from PCA	96%	training needed	laptop with Intel 5300 and TP-link TLWDR7500
WiGest [43]	Gestures: swipe left/right, open/close, wipe, hold over, toward	RSSI-based, segmentation and matching	87.5%	no training	two routers used in the experiment were the Cisco Linksys X2000 and the Netgear N300
Device-free occupant activity sensing using Wi-Fi-enabled IoT devices for smart homes [15]	Activities: run, jump, sit down, box, walk, golf	Fine-grained CSI-based, CSVD-NMF classification with KNN	90.6% with 20 training samples	training needed	TP-Link N7500 along with other COTS Wi-Fi routers
Freedetector [16]	Occupied or unoccupied	CSI-based, classification with random forest	93.73%	training needed	two TP-LINK N750 wireless dual-band routers
Deepsense [26]	Sitting, standing, walking, lying down, and running	CSI-based, classification with CNN	97.4%	training needed	two TP-LINK N750 wireless routers
Fine-grained occupant activity monitoring with Wi-Fi channel state information [17]	Activities: cooking, eating, fall down, toileting, sleeping, walking, batching, empty	CSI-based, feature extraction and classification with CNN-LSTM model	96% for one user	training needed	four Lenovo T400 laptops equipped with Intel 5300NIC, where one laptop served as the transmitter and three laptops were employed as receivers
Using auditory features for Wi-Fi channel state information activity recognition [18]	Activities: walking, empty, pushing, waving, boxing	CSI-based, classification with CNN classifier combined with MFCC features	95%, 88.64%, and 78% for CARM-9, CARM-11, ITI dataset respectively	training needed	TP-LINK: AC-1750 used as transmitter desktop computer with the Intel-5300 Wi-Fi card used as receiver
CDHAR [19]	Activities: walk, run, sit down, squat, and fall down	Fine-grained CSI-based, classification with ensemble learner	90% indoor and 91% outdoor	training needed	MS-B083 mini host equipped with an Intel 5300 NIC; both the transmitter and receiver were configured with a single antenna
Device-free activity recognition using ultra-wideband radios [20]	Activities: standing, sitting, lying	UWB radios, classification with random forest	95.6%	training needed	UWB (ultra-wideband) transceivers and UWB transmitter; we utilized the DW1000 Evaluation Board (EVB1000) provided by DecaWave
FALAR [22]	Activities: walk, run, sit down, boxing, and golf swinging	Fine-grained CSI-based, classification with CSVD-NMF	90%	training needed	two TP-LINK N750 wireless dual-band routers

**Table A3:** Comparison between Wi-Fi-based system of human movement detection.

System	Objective of Sensing	Methodology	Performance	Complexity	Devices
WiWho [14]	Gait of single person	CSI-based, a decision tree-based classifier	80% with 6 people	training needed	a laptop equipped with an Intel 5300 NIC and Asus RT-AC66U routers
A Trained-once Crowd Counting Method Using Differential Wi-Fi Channel State Information [46]	Count the number of people in each area at most for seven people	CSI-based, classification using the linear discriminant classifier	74% for Room A (5 m × 6 m) and 52% for Room C (6 m × 12.5 m).	training needed	a commercially available 2.4 GHz Wi-Fi b/g/n access point (AP) was utilized as a dual-antenna transmitter; additionally, a laptop running Ubuntu 10.04 LTS equipped with an Intel Wi-Fi Link 5300 wireless NIC, featuring three antennas, served as the receiver
WiDMove [44]	Human direction detection	CSI-based, classification using SVM	80%	training needed	two computers were utilized in the experiment, each equipped with an Intel Core i7 processor, 8 GB of RAM, and running Ubuntu 14.04; both computers were also equipped with Atheros ath9k AR9380 NICs
WifiU [45]	Walking movement	CSI-based, PCA, SVM classifiers	79.28%/89.52%/93.05% (top-1/-2/-3, 50 subjects)	training needed	NetGear JR6100 Wi-Fi router and a ThinkPad X200 laptop equipped with an Intel 5300 Wi-Fi NIC

**Table A4:** Comparison between Wi-Fi-based system of human walking direction detection.

System	Objective of Sensing	Methodology	Performance	Complexity	Devices
WiDir [9]	Detect walking direction	CSI-based, Fresnel direction calculation	median error of less than 10 degrees	no training needed	a Wi-Fi access point (AP) and two computers equipped with wireless cards
WiDar [10]	Detect human velocity (direction and speed) and location	CSI Doppler, STFT Gaussian window, pass band filter PCA	the median location error observed is 25 cm and 38 cm when considering both cases with and without initial positions; additionally, a median relative velocity error of of 13%	no training	three mini-desktops equipped with Intel 5300 NICs; one of the mini-desktops was designated as the transmitter, while the other two were employed as receivers
WiDet [31]	Walking direction detection (up/down)	RSSI-based, CNN algorithm	94.5% for a total of 163 walking events	training needed	the system operates on a Raspberry Pi development board, running on the Linux kernel version 2.6. It comprises three Wi-Fi transmitters and a single Wi-Fi receiver
Wi-Fi-Enabled Smart Human Dynamics Monitoring [32]	Counting people, walking direction detection	RSSI-based, CNN algorithm	90%	training needed	two sets of laptops, with each pair equipped with Intel 5300 802.11n Wi-Fi NICs, functioning as Wi-Fi transceivers
Indoor Device-Free Activity Recognition Based on Radio Signal [33]	Walking direction detection	PRR-based, KNN and SVM classifier	91%	training needed	four transmitters USRP nodes and one receiver
Walking Direction Detection using Received Signal Strengths in Correlated RF Links [34]	Walking entry and exit directions	RSSI-based, dynamic time warping (DTW)	the percentage ranges from 40% to 99%, depending on the specific pair of links utilized	training needed	the XM2110 node, developed by MEMSIC Inc.; this node combines an ATmega1281 processing chip with a mote module that supports the IEEE 802.15.4 protocol
Gate-ID [35]	User’s gait in two walking directions (left, right)	CSI-based, deep learning algorithms	90.7%	training needed	an HP laptop and a Netgear R7000 Wi-Fi router
WiDIGR [36]	Walking direction detection	CSI-based, SVM classifier	78.28% for apartment, 92.83% for empty room for training data	training needed	three laptops equipped with Intel 5300 wireless NICs
Our Approach	Walking direction detection (left, right, up, down) and empty	CSI-based, SVM classifier	92.9%, 95.1%, and 89% for untrained data of the classroom, meeting room, and classroom + meeting room, respectively	training needed	two TP-LINK TLWDR4300 wireless routers and a single-user laptop
